# Ectopic Cushing’s syndrome secondary to olfactory neuroblastoma

**DOI:** 10.1007/s00701-017-3447-y

**Published:** 2018-01-17

**Authors:** Kenny Yu, Federico Roncaroli, Tara Kearney, David Ewins, Deepa Beeharry, Thomas Naylor, David Ray, Rajiv Bhalla, Kanna Gnanalingham

**Affiliations:** 1 Department of Neurosurgery, Salford Royal NHS Foundation Trust, Manchester Academic Health Science Centre, Salford, M6 8HD UK; 2 Division of Neuroscience and Experimental Psychology, Faculty of Biology, Medicine and Health, School of Biological Science, University of Manchester, Manchester, M13 9PT UK; 3 Department of Endocrinology, Salford Royal NHS Foundation Trust, Manchester Academic Health Science Centre, Salford, M6 8HD UK; 4 Department of Endocrinology, Countess of Chester Hospital, Liverpool Road, Chester, CH2 1UL UK; 5 Department of Otorhinolaryngology, Salford Royal NHS Foundation Trust, Manchester Academic Health Science Centre, Salford, M6 8HD UK

**Keywords:** Olfactory neuroblastoma, Ectopic, ACTH, Cushing’s

## Abstract

We present the case of a patient with Cushing’s syndrome secondary to ectopic ACTH secretion. A MR of the head showed a left-sided nasal mass extending down from the cribriform plate. The patient underwent endoscopic resection with nearly complete removal of the mass. Histological examination showed an ACTH-secreting olfactory neuroblastoma (ONB). The patient’s cortisol levels returned to normal range after surgery and have remained normal for over a year. ONB is a rare cause for ectopic ACTH secretion. This case highlights the diagnostic and management difficulties in patients with ectopic ACTH secretion, and provides a brief review of ONB.

## Case presentation

A previously healthy 55-year-old gentleman presented with a 1-year history of progressive sore throat, post nasal drip, excessive fatigue, generalised muscle weakness and reduced exercise tolerance. He had noted increased truncal weight gain and difficulty climbing stairs, culminating in a fall and hospitalisation.

On examination, he had truncal obesity, poor skin healing, bruising around venepuncture sites, bilateral pitting oedema up to the sacrum and signs of proximal myopathy. His blood pressure on admission was elevated at 180/95 mm/Hg. He had intermittent episodes of confusion and was noted to have lost his sense of smell and taste.

Investigations revealed marked hypokalaemic alkalosis that was initially resistant to treatment with enalapril, spironolactone and Sando-K (serum potassium 2.5 mmol/l [normal range 3.5–5.0 mmol/l], serum bicarbonate 45 mmol/l [normal range 20–32 mmol/l]). Although not previously known to be diabetic (HbA1c 3 months prior to admission was just 45 mmol/mol), a random blood glucose on admission was elevated at 28.6 mmol/l and his HbA1c was 71 mmol/mol, confirming recent onset diabetes. As he was symptomatic with thirst and polyuria, he was started on Humulin I insulin (20 units b.d.).

A diagnosis of Cushing’s syndrome was made and in view of the rapid onset of symptoms with marked metabolic features and fluid retention, the possibility of ectopic ACTH was entertained.

Random cortisol (2213 nmol/l), adrenocorticotrophic hormone (ACTH) (40 pmol/l) and 24-h urine free cortisol (18,091 nmol/24 h) levels were significantly elevated [normal ranges < 607 nmol/l, 2–11 pmol/l and < 165 nmol/24 h, respectively]. A subsequent dexamethasone suppression test failed to demonstrate adequate suppression on low dose (cortisol 1562 nmol/l, ACTH 76 pmol/l) or high dose dexamethasone (cortisol 1625 nmol/l, ACTH 59 pmol/l). These results confirmed ACTH-dependent Cushing’s syndrome.

**Fig. 1:**
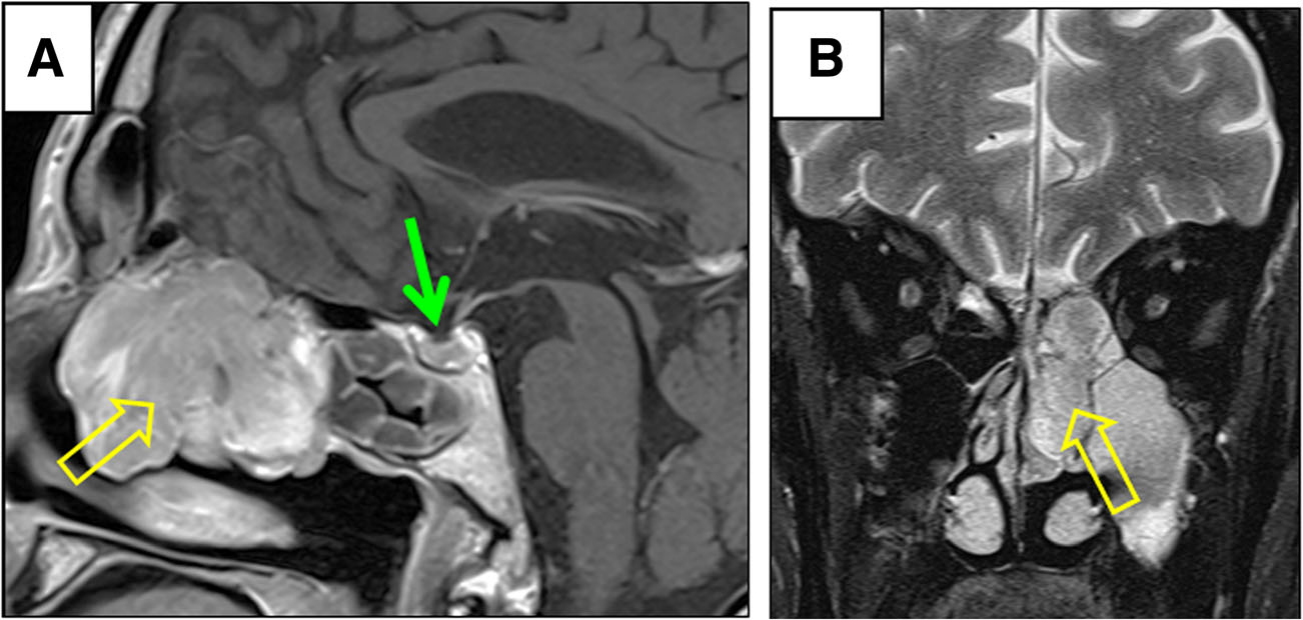
Sagittal T1-weighted MRI with gadolinium contrast (**a**) and Coronal T2-weighted MRI (**b**) revealing an enhancing nasal mass extending down from the cribriform plate of the anterior cranial fossa (open arrow), with the presence of a normal pituitary gland (solid arrow)

MR scan of brain revealed a normal pituitary gland (Fig. 1a, solid arrow), with no asymmetry in contrast enhancement. However, the scan also revealed a left-sided nasal mass (Fig. 1a, open arrow) extending down from the cribriform plate and involving the ethmoid sinuses and the ipsilateral turbinates. The lesion spanned from the back of frontal sinus to just anterior to the sphenoid sinus.

Whole body CT scan revealed bilateral nodular hyperplasia of the adrenal glands and no other mass lesions and the patient underwent a diagnostic endoscopic biopsy.

Subsequent testing revealed a high ACTH precursor concentration of 613 pmol/l [normal range 0–40 pmol/l] suggestive of an ectopic source of ACTH [4].

Medical management of the ectopic ACTH syndrome proved difficult with maximally tolerated doses of metyrapone (750 mg t.d.s.) and it was only after the addition of ketoconazole (800 mg daily t.d.s) that his general health and cortisol profile (474–599 nmol/l) improved sufficiently to allow surgery.

The patient underwent endoscopic resection via an endonasal approach, with a right naso-septal flap hinged on the sphenopalatine pedicle for subsequent repair. A moderately soft and avascular left-sided nasal tumour arising from the cribriform plate was resected in piecemeal fashion. The adjacent cribriform plate was partly eroded; however, the overlying dura mater was intact. All abnormal tissue down to the cribriform plate was resected, with diathermy of the anterior skull base dura, which was left largely in situ to minimise the risk of post-op CSF leak. Nasal septal flap was then used to cover the anterior skull base dura, supported with fibrinogen sealant (i.e., Tisseel, Baxter, UK), Nasopore (Stryker, UK) and a Foley balloon catheter for 7 days.

**Fig. 2:**
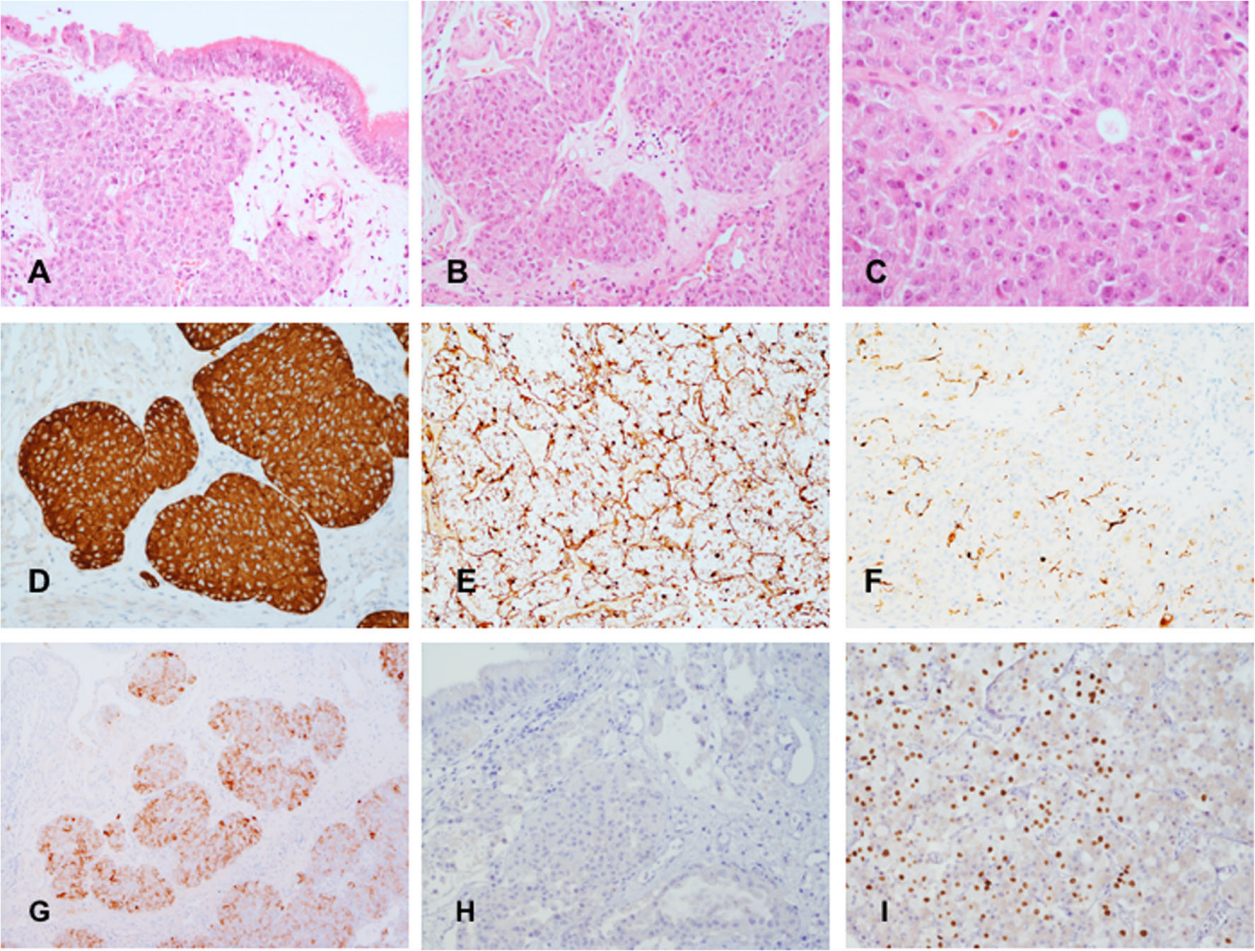
Nasal mucosa appears to be infiltrated by tumour (**a** haematoxylin and eosin, ×10); the lesion has lobular and sheet-like architecture (**b** haematoxylin-eosin, ×10) and consists of moderately atypical cells with scanty cytoplasm (**c** haematoxylin-eosin, ×20). Neoplastic cells are intensely positive for synaptophysin (**d** immunoperoxidase, ×10); the immunoreaction for S-100 protein highlights sustentacular cells (**e** immunoperoxidase, ×20); the present of focal neuropil is shown with the immunoreaction for neurofilament proteins (**f** immunoperoxidase, ×20); several tumour cells are positive for ACTH (**g** immunoperoxidase, ×4); there is no expression of T-pit (**h** immunoperoxidase, ×20); normal adenohypophysis used as control shows normal T-pit expression (**i** immunoperoxidase, ×20)

Tissue from the initial transnasal biopsy and from endoscopic resection showed respiratory-type mucosa and bone infiltrated by a tumour composed of sheets and merging lobules. Lobules were delimited by fibro-vascular connective tissue. Neoplastic cells had scanty cytoplasm and moderately atypical nucleus with fine, salt and pepper chromatin and small nucleolus. Mitoses were rare (average 2 × 10 high power fields at × 40 magnification). Necrosis was absent. A few Homer-Wright pseudo-rosettes were present. Tumour cells were intensely and uniformly positive for neuron specific enolase and synaptophysin. Chromogranin A was focally expressed immunostains for cytokeratin 7, cytokeratin 20, cytokeratin CAM5.2 were negative. The immunostain for S-100 protein revealed a meshwork of sustentacular cells and the immunoreaction for neurofilament proteins documented focal neuropil. No obvious neuronal differentiation was noted. Immunostains for pituitary hormones showed ACTH expression in several neoplastic cells but neoplastic cells did not express the transcription factor T-pit (courtesy Dr. Olivera Casar-Borotra, Clinical Uppsala University Hospital Uppsala, Sweden) (Fig. 2). Pathological features were consistent with low-grade (Hyams’ grade II) olfactory neuroblastoma (ONB) with ectopic ACTH. About 2% of neoplastic cells were positive for Ki-67. Absence of cytokeratin CAM5.2 expression, the presence of sustentacular cells, focal expression of neurofilament protein and the absence of T-pit expression excluded the possibility of an ectopic corticotroph adenoma.

Post-operative recovery was slow. There was rapid normalisation of serum glucose and blood pressure. Anti-hypertensive medications, insulin, metyrapone and ketoconazole were withdrawn. This coincided with improvement in his early morning cortisol levels, without steroid replacement. An overnight dexamethasone suppression test at 4 weeks following surgery revealed an adequate suppression of early morning cortisol to 14 nmol/l, consistent with remission of his Cushing’s syndrome. The patient proceeded to have adjuvant radiotherapy to the post resection cavity and remains in remission at 1-year follow-up.

## Discussion

Cushing’s disease secondary to ACTH-secreting pituitary adenoma is the most common cause of Cushing’s syndrome. Other causes include iatrogenic steroids and adrenal adenomas. Ectopic secretion of ACTH accounts for about 10–15% of cases of hypercortisolism [5] and it is usually associated with lung and pancreatic neuroendocrine tumours.

Ectopic ACTH was suspected in this case due to the rapid onset of symptoms and the marked metabolic features, lack of suppression after dexamethasone and normal pituitary at MRI.

ONB accounts for 2–3% of tumours of the nasal cavity and it is likely derived from the olfactory basal reserve cells in the olfactory mucosa [1]. ONB occurs in a wide range of ages and frequently presents with nasal obstruction, epistaxis and anosmia [7]. The prognosis of ONB is variable with 10-year survival rates between 45 and 76% [6]. Adjuvant radiotherapy for the primary lesion site was given in this case to reduce the risk of local recurrence [8]. Long-term follow-up of these patients is recommended. Rarely ONB can cause a paraneoplastic syndrome that is secondary to ectopic secretion of peptides and hormones, or an immune response due to cross-reactivity between neoplastic cells and normal tissues [3]. Endocrinological manifestations of ONB include syndrome of inappropriate ADH secretion, ectopic ACTH syndrome, humoral hypercalcemia of malignancy, hypertension caused by catecholamine secretion and hyperprolactinemia [2]. Only four cases with ACTH secretion confirmed at immunohistochemistry on tissue have previously been reported [1].

Identifying the source of ACTH in ACTH-dependent Cushing’s syndrome can be challenging. The MR scan of the pituitary, even with dynamic contrast enhancement does not always reveal an adenoma [3]. In such cases, CT scan of body (for ectopic source) and inferior petrosal venous sinus sampling (IPSS) may help in the localisation of ACTH source [3]. The presence of high levels of ACTH precursor molecules can also suggest an ectopic source of ACTH [4]. However, in the present case, the proximity of ONB to the pituitary gland and the potential sharing of venous drainage may have led to false positive results with IPSS. Hence, surgical biopsy of the nasal lesion was helpful in the diagnosis and guided subsequent treatment. Although surgical resection of the primary ACTH-producing lesion can normalise cortisol levels, surgical cure in rare.

The present case highlights some of the diagnostic challenges in isolating the source of ACTH production and the surgical management of the condition.
